# Result of the Serum Treatment of Diphtheria in Russia

**Published:** 1898-12

**Authors:** 


### Result of the Serum Treatment of Diphtheria in Russia.

In the Russian medical paper *"Medicinskoe Obosrenie,"* Vol. 50, November 3, 1898, Dr. Wertepow gives some very interesting details of the anti-diphtheric serum in the "Staniza (Colony of Kossacks) Slepzowskaja." The same number contains a highly interesting case of a very late application of the serum with complete recovery, written by Dr. Bronstein.

1. W. reports on his experience with the serum in a late diphtheria epidemic.

Of those patients who were treated with the serum, nine in all, 5 per cent. died; the other patients who were treated by other means showed a mortality of 40 per cent.

2. The interesting case of Dr. B. was a girl, *aet.* 3, suffering from a very grave diphtheritis of the fauces with transition to the larynx.

Unfortunately there was no fresh serum at hand, and Dr. B. treated the case by all the known remedies, but without success. Meanwhile the state of the girl became worse and worse, and had soon to be considered hopeless.

Then Dr. B. resolved, on the sixth day of the disease, to apply a serum one and one-half years old. It was already turbid, but on shaking it, it showed neither flakes nor filaments. Dr. B. made two injections, altogether of the strength of 1.1000, and the result was an astonishing successful one. All local phenomena diminished and gradually retrogressed, the general state of the little sufferer became better and better, and the child recovered completely.

A remarkable effect in another direction took place as well. The child had suffered, previously to the attack of diphtheria, from a very obstinate eczema, which, after the application of the serum, disappeared completely.—*N. O. Med. Journal.*

---

**Death in Crape.**—"Many a woman has been laid in her coffin by wearing of crape," writes a physician. "It is a sin to do or wear anything that hurts the health, and therefore I think it positively sinful for women to wear mourning. Even plain black is not wholesome. It is astonishing that this custom has not been abolished, for women have grown very sensible in the matter of dress. It would have been abolished long ago were it not for the fact that woman cares more for what other people say than she does for herself. For example, if a woman's husband dies she would not dare to go without a long crape veil and a crape trimmed gown, or the world would say she was glad to get rid of him; after wearing it awhile she is not brave enough to leave it off, for she knows the same relentless world will say she is looking for number two. Women claim that mourning is a protection. If one is really grief-stricken one's own feelings are sufficient protection against society, and for my part I believe that crape and other mourning habiliments are often directly responsible for bad complexion, bad eyes, bad digestion, and bad temper.—*Dietetic and Hygienic Gazette.*

